# Distal His bundle pacing in a patient with surgically corrected complex Ebstein anomaly and symptomatic second-degree atrioventricular block: a case report

**DOI:** 10.1093/ehjcr/ytad531

**Published:** 2023-10-31

**Authors:** Ivelin Koev, G Andre Ng, Aidan P Bolger, Mokhtar Ibrahim

**Affiliations:** Department of Cardiovascular Sciences, University of Leicester, University Rd, Leicester LE1 7RH, UK; Department of Cardiology, Glenfield Hospital, University Hospitals of Leicester NHS Trust, Groby Rd, Leicester LE3 9QP, UK; Department of Cardiovascular Sciences, University of Leicester, University Rd, Leicester LE1 7RH, UK; Department of Cardiology, Glenfield Hospital, University Hospitals of Leicester NHS Trust, Groby Rd, Leicester LE3 9QP, UK; National Institute for Health Research Leicester Biomedical Research Centre, Leicester, UK; Department of Cardiovascular Sciences, University of Leicester, University Rd, Leicester LE1 7RH, UK; National Institute for Health Research Leicester Biomedical Research Centre, Leicester, UK; East Midlands Congenital Heart Centre, Glenfield Hospital, University Hospitals of Leicester NHS Trust, Leicester, UK; Department of Cardiology, Glenfield Hospital, University Hospitals of Leicester NHS Trust, Groby Rd, Leicester LE3 9QP, UK

**Keywords:** Conduction system pacing, His pacing, Ebstein’s anomaly, 2nd-degree Mobitz II AV block, Right bundle branch block, Case report

## Abstract

**Background:**

Ebstein’s anomaly occurs when there is an apical displacement of the tricuspid valve with septal and posterior valve leaflets tethering. This condition often occurs in association with other congenital, structural, or conduction system diseases, including intracardiac shunts, valvular lesions, arrhythmias, accessory conduction pathways, and first-degree atrioventricular (AV) block. We present for the first time a case of a patient with Ebstein’s anomaly who presented with second-degree Mobitz II AV block and was successfully treated with conduction system pacing (CSP) due to her young age and the likelihood of a long-term high percentage of pacing.

**Case summary:**

We present a case of a 42-year-old lady with a background of complex congenital heart disease, including severe pulmonary stenosis, Ebstein anomaly, and atrial septal defect (ASD). She required complex surgical intervention, including tricuspid valve (TV) repair and subsequently replacement, ASD closure, and pulmonary balloon valvuloplasty. She presented to our hospital with symptomatic second-degree Mobitz II AV block (dizziness, shortness of breath, and exercise intolerance) and right bundle branch block (RBBB) on her baseline ECG. Her echocardiogram showed dilated right ventricle (RV) and left ventricle (LV) with low normal LV systolic function. Due to her young age and the likelihood of a long-term high percentage of RV pacing, we opted for CSP after a detailed discussion and patient consent. The distal HIS position is the preferred pacing strategy at our centre. We could not cross the TV with the standard Medtronic C315 HIS catheter, so we had to use the deflectable C304 HIS catheter. Mapping and pacing of the distal HIS bundle were achieved by Medtronic Selectsecure 3830, 69 cm lead. HIS bundle pacing led to the correction of both second-degree Mobitz II AV block and pre-existing RBBB. The implantation was uneventful, and the patient was discharged home the next day without any acute complications.

**Discussion:**

Distal HIS pacing is feasible in patients with surgically treated complex Ebstein anomaly and heart block. This approach can normalize the QRS complex with a high probability of preserving or improving LV function.

**Learning points**

- Crossing the tricuspid valve could be problematic, so that a deflectable C315 HIS catheter can facilitate the process.
- Distal HIS pacing is achievable and safe in patients with surgically treated complex Ebstein’s anomaly and heart block.
- This approach can normalize the QRS complex with a high probability of preserving or improving LV function.

## Introduction

Conduction system pacing (CSP) gained popularity in the last decade as a more physiological alternative to right ventricular pacing for treating bradycardia or as an alternative to biventricular pacing for cardiac resynchronization therapy (CRT). It is divided into His bundle pacing (HBP) and left bundle branch area pacing. The current European Society of Cardiology guidelines for pacing and CRT present HBP as a class IIb indication (level of evidence C) and a left ventricular ejection fraction > 40% who require frequent (more than 20%) ventricular pacing or as part of a ‘pace and ablate’ strategy for rapidly conducted supra-ventricular arrhythmias. It also has a class IIa indication (level of evidence B) in CRT candidates in case of failed coronary sinus lead implantation.^1^ In patients with narrow QRS complexes, His bundle pacing allows similar ventricular activation pattern with preservation of QRS duration and morphology.^2^

To the best of our knowledge, we report the first case of HBP in a patient with corrected Ebstein’s anomaly, second-degree Mobitz II AV block, and RBBB.

## Summary figure

**Figure:**
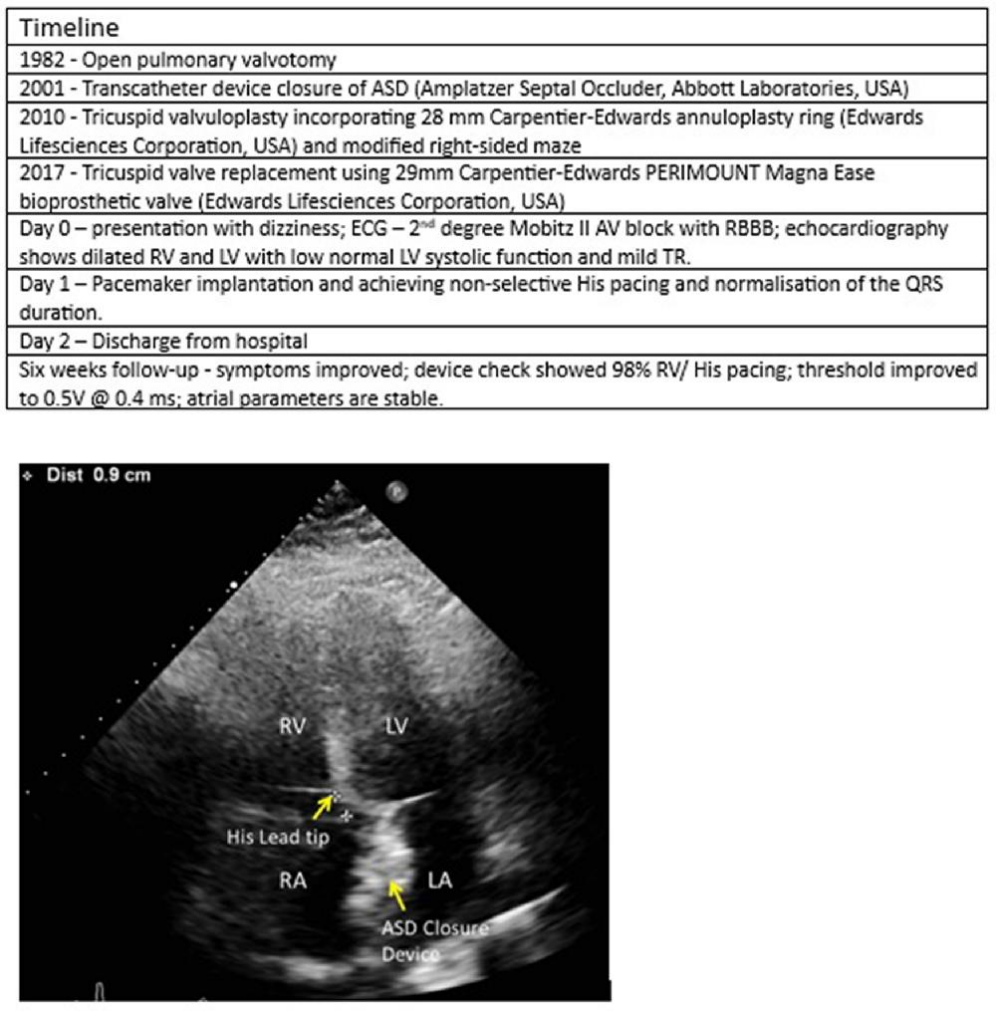


## Case summary

**Figure 1:**
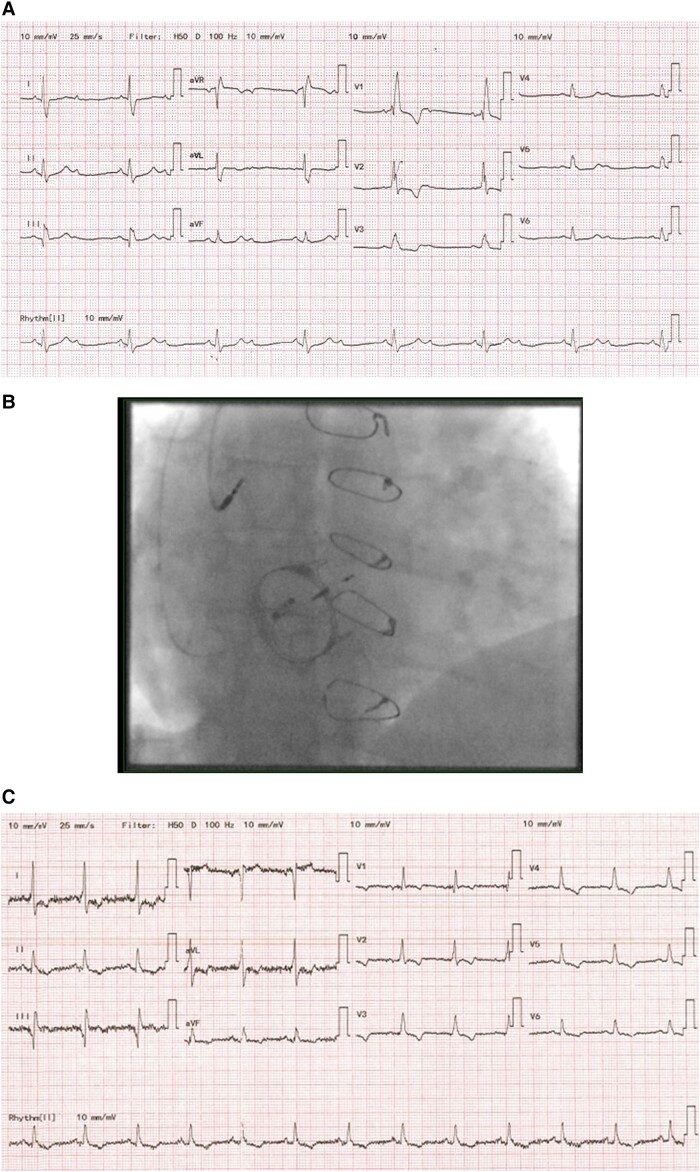
(*A*) 12-Lead ECG on admission. (*B*) Antero-posterior view on X-ray after pacemaker implantation. (*C*) 12-Lead ECG after His bundle pacemaker implantation.

We present a case of a 42-year-old lady with a background of complex congenital heart disease, including severe pulmonary stenosis, Ebstein anomaly, and atrial septal defect (ASD). She required open pulmonary valvotomy in 1982, transcatheter device closure of ASD (Amplatzer Septal Occluder, Abbott Laboratories, USA), tricuspid valvuloplasty incorporating 28 mm Carpentier-Edwards annuloplasty ring (Edwards Lifesciences Corporation, USA), and modified right-sided maze and tricuspid valve replacement using 29 mm Carpentier-Edwards PERIMOUNT Magna Ease bioprosthetic valve (Edwards Lifesciences Corporation, USA). Her medical history also includes heterozygous factor V Leiden deficiency, benign intracranial hypertension and previous vitreous haemorrhage with bilateral papilloedema. She presented to our hospital with newly developed dizziness and previously reported shortness of breath and exercise intolerance. Her resting ECG showed a right bundle branch block, QRS of 152 ms, 2:1 heart block, and normal QRS axis (*Figure 1A*). Lung sounds were clear bilaterally in all lobes anteriorly and posteriorly. There was a variation in the intensity of the first heart sound consistent with a 2:1 atrioventricular (AV) block. Vital signs were stable, and laboratory tests did not show any abnormality.

Her echocardiogram showed dilated RV and LV, low normal LV systolic function, and mild tricuspid regurgitation. This represented a worsening in her LV function, which was normal on previous exams. Due to her young age and the likelihood of a long-term high percentage of RV pacing, we opted for CSP after a detailed discussion and consent from the patient.

Ultrasound-guided axillary venous access was obtained for both atrial and CSP lead. A Medtronic SelectSecure 3830 lead, 69 cm lead, and C315 HIS catheter were used to map and pace the HIS bundle. We encountered difficulties crossing the TV, so we used the deflectable C304 HIS catheter. The distal HIS position is the preferred pacing strategy at our centre. An anatomical approach was targeted at the basal RV septal site after crossing the prosthesis with a small HIS, far-field, and a large ventricular signal (*Figure 1B*).

We managed to achieve full correction of right bundle branch block (RBBB) with non-selective HIS pacing throughout. Pacing parameters were satisfactory with sensing of 8 mV, threshold of 1 mV at 0.4 ms, and local RV capture of 0.375 V at 0.4 ms with a paced QRS of 114 ms (*Figure 1C*). The patient was discharged home the next day without any acute complications.

She came for her device follow-up 6 weeks post-implantation. Symptoms improved, the device check showed 98% RV/HIS pacing, and threshold improved to 0.5 V at 0.4 ms. Atrial parameters are stable. There is a good biventricular function and trivial tricuspid regurgitation on echocardiography.

## Discussion

Ebstein’s anomaly occurs when there is an apical displacement of the tricuspid valve with septal and posterior valve leaflets tethering. This condition often occurs in association with other congenital, structural, or conduction system diseases, including intracardiac shunts, valvular lesions, arrhythmias, accessory conduction pathways, and first-degree AV block.^3^ High-degree AV block is a rare presentation.^4^ Tricuspid valve surgeries and challenging morphological abnormalities in these patients often demand exceptional skills during pacemaker implantation.^5^ Sharma *et al.*^6^ demonstrated the feasibility of HBP in patients with RBBB allowing further widening the potential benefits of CSP. His bundle pacing can significantly benefit the physiological ventricular activation pattern. Nevertheless, it presents challenges such as the difficulty of lead implantation, reduced R-wave amplitudes, and high and unstable pacing thresholds. His bundle pacing in patients with Ebstein’s anomaly could be even more challenging as in some cases, His-potentials could be demonstrated only in the atrialized portion of the ventricle^7^ or because of a possible intra- and infra-hisian conduction delay.^8^

In this case, the HBP was confirmed by as follows:

1. HIS signal on the lead intracardiac electrogram.
2. Two different thresholds on threshold testing; first at 1 V with correction of the RBBB and second is local RV septal capture at a lower output, broader QRS notched, and LBBB morphology.

We achieved full correction of RBBB with non-selective HIS pacing accompanied by the disappearance of the patient’s symptoms.

The effect of transvalvular lead placement on tricuspid regurgitation in patients with tricuspid valve repair or replacement is an important factor to be considered before the decision for pacemaker implantation. Cardiovascular implantable electronic device-related tricuspid regurgitation due to lead placement is a possible complication of pacemaker implantation secondary to direct trauma or further interaction of the electrode leading to impingement of the leaflets and malcoaptation. In addition, leaflet adhesion, fibrosis, and encapsulation could contribute to tricuspid regurgitation.^9^ Alternatives to transvalvular lead implantation in patients with tricuspid valve replacement are described in the literature. Lead placement in the lateral and anterolateral veins also provides the benefit of resynchronization and presents good lead parameters.^10–12^ Paravalvular lead placement is achievable^13^ as well as leadless permanent pacemaker implantation.^14^

In conclusion, pacemaker implantation is challenging in patients with surgically treated complex Ebstein’s anomaly and heart block. We demonstrated that distal HIS pacing is feasible in these patients. This approach can normalize the QRS complex with a high probability of preserving or improving LV function.

## Supplementary Material

ytad531_Supplementary_Data

## Data Availability

The data underlying this article are available in the article and in its online Supplementary material.

## Lead author biography

Dr Ibrahim graduated from Ain Shams University in Egypt in 2005. He works as a consultant cardiologist and electrophysiologist at the University Hospitals of Leicester, UK. He established conduction system pacing in Leicester and leading this service nationally with education, training, and research.
