# Supplementary figures and images for: Distal His bundle pacing in a patient with surgically corrected complex Ebstein anomaly and symptomatic second-degree atrioventricular block: a case report
Source: Eur Heart J Case Rep. 2023 Oct 31;7(12):ytad531. doi: 10.1093/ehjcr/ytad531 (PMC10691874; doi:10.1093/ehjcr/ytad531)

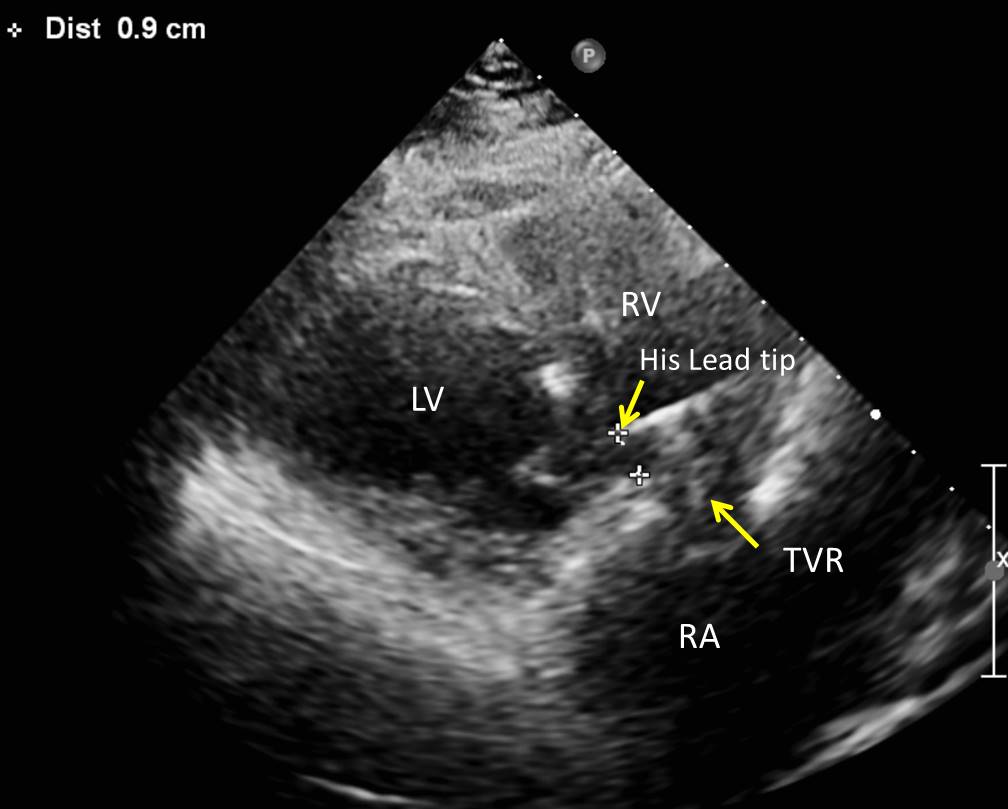

Supplement: ytad531_Supplementary_Data [file ytad531_supplementary_data.zip › Modified PLAX view His lead.jpg]

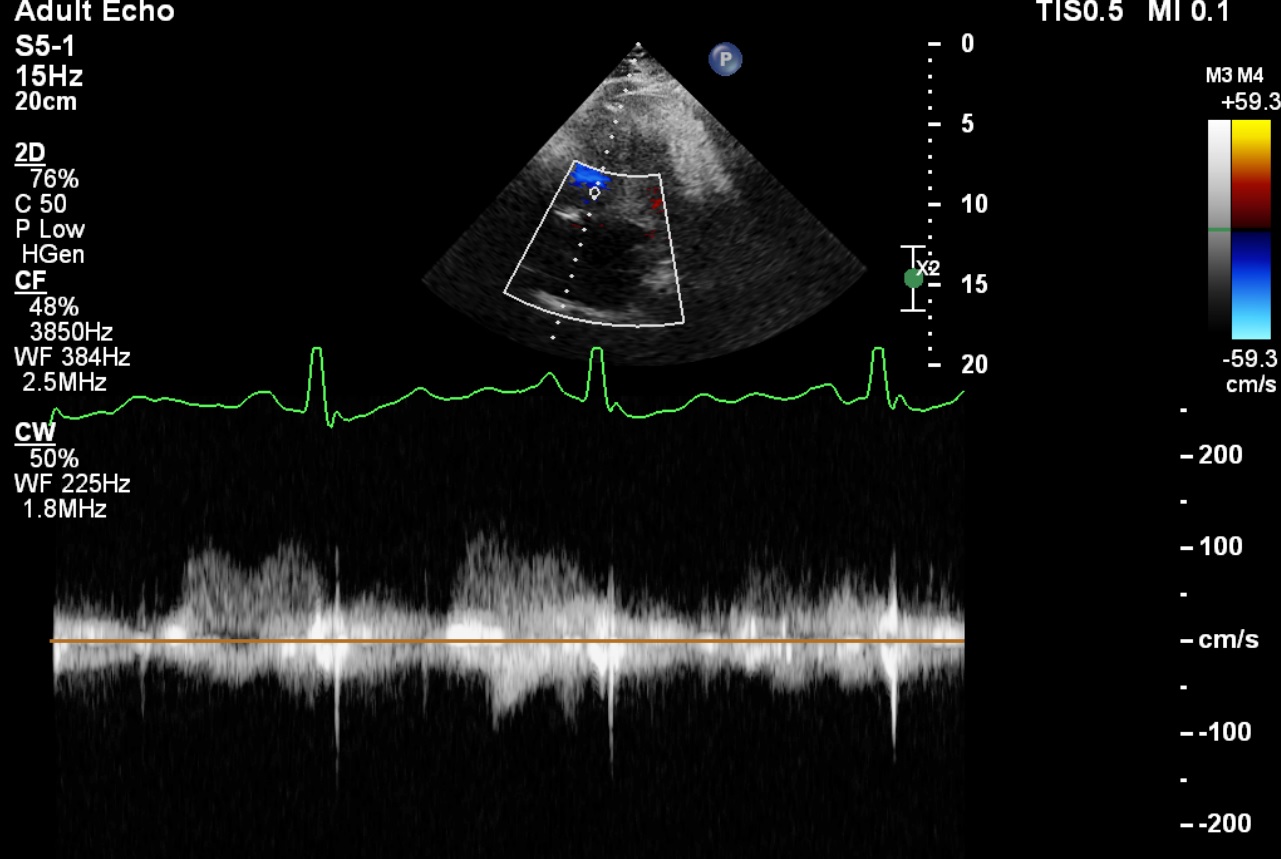

Supplement: ytad531_Supplementary_Data [file ytad531_supplementary_data.zip › echo 4C Tricuspid valve.jpg]

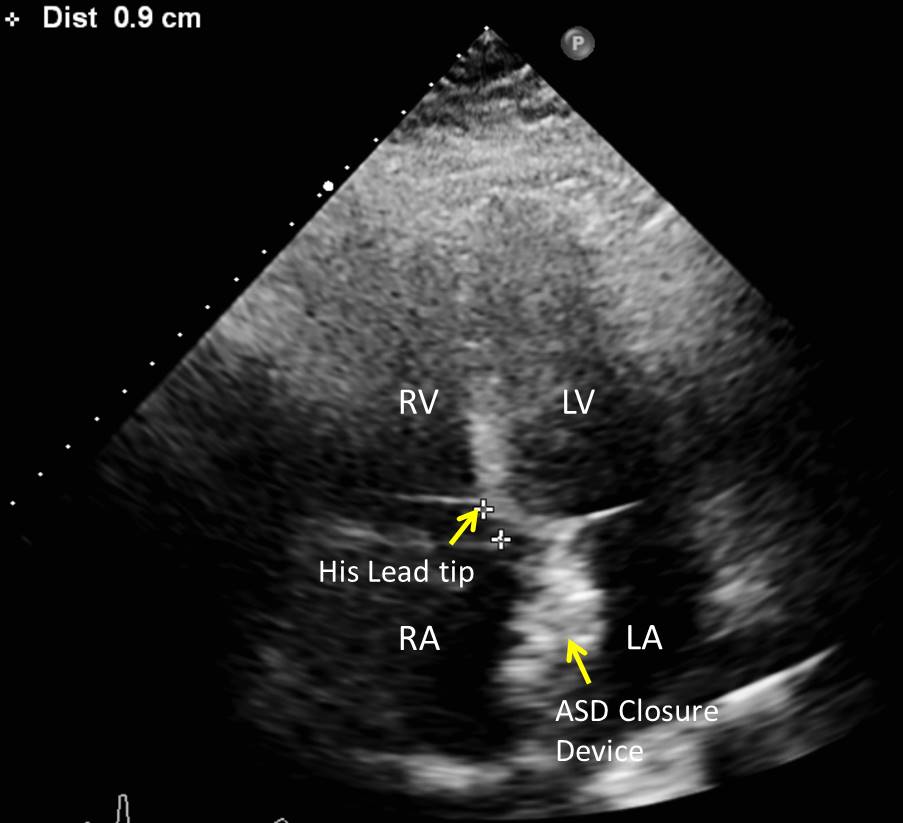

Supplement: ytad531_Supplementary_Data [file ytad531_supplementary_data.zip › Echo apical 4C view.jpg]
